# Pneumocafé project: an inquiry on current COPD diagnosis and management among General Practitioners in Italy through a novel tool for professional education

**DOI:** 10.1186/2049-6958-9-35

**Published:** 2014-06-12

**Authors:** Claudio M Sanguinetti, Fernando De Benedetto, Claudio F Donner, Stefano Nardini, Alberto Visconti

**Affiliations:** 1 Consultant in Respiratory Medicine and CEO of FISAR, Rome, Italy; 2 Pneumology Department, SS Annunziata Hospital, Chieti, Italy; 3 Mondo Medico, Multidisciplinary and Rehabilitation Outpatient Clinic, Borgomanero, NO, Italy; 4 Pulmonary and TB Unit-Vittorio Veneto (TV), Vittorio Veneto, Italy; 5 FISAR ICT Engineer and Consultant, Arona (NO), Italy

**Keywords:** COPD diagnosis, Early diagnosis, General practice, GPs, Respiratory specialist, Spirometry

## Abstract

**Background:**

Symptoms of COPD are frequently disregarded by patients and also by general practitioners (GPs) in early stages of the disease, that consequently is diagnosed when already at an advanced grade of severity. Underdiagnosis and undertreatment of COPD and scarce use of spirometry are widely recurrent, while a better knowledge of the disease and a wider use of spirometry would be critical to diagnose more patients still neglected, do it at an earlier stage and properly treat established COPD. The aim of Pneumocafè project is to improve, through an innovative approach, the diagnosis and management of COPD at primary care level increasing the awareness of issues pertaining to early diagnosis, adequate prevention and correct treatment of the disease.

**Methods:**

Pneumocafè is based on informal meetings between GPs of various geographical zones of Italy and their reference respiratory specialist (RS), aimed at discussing the current practice in comparison to suggestions of official guidelines, analyzing the actual problems in diagnosing and managing COPD patients and sharing the possible solution at the community level. In these meetings RSs faced many issues including patho-physiological mechanisms of bronchial obstruction, significance of clinical symptoms, patients’ phenotyping, and clinical approach to diagnosis and long-term treatment, also reinforcing the importance of a timely diagnosis, proper long term treatment and the compliance to treatment. At the end of each meeting GPs had to fill in a questionnaire arranged by the scientific board of the Project that included 18 multiple-choice questions concerning their approach to COPD management. The results of the analysis of these questionnaires are here presented.

**Results:**

1, 964 questionnaires were returned from 49 RSs. 1,864 questionnaires out of those received (94.91% of the total) resulted properly compiled and form the object of the present analysis. The 49 RSs, 37 males and 12 females, were distributed all over the Italian country and practiced their profession both in public and private hospitals and in territorial sanitary facilities. GPs were 1,330 males (71.35%) and 534 females (28.64%), mean age 56,29 years (range 27-70 yrs). Mean duration of general practice was 25.56 years (range: 0,5-40 yrs) with a mean of 1,302.43 patients assisted by each GP and 2,427,741 patients assisted in all. The majority of GPs affirmed that in their patients COPD has a mean-to-great prevalence and a mean/high impact on their practice, preceded only by diabetes and heart failure. Three-quarters of GPs refer to COPD guidelines and most of them believe that a screening on their assisted patients at risk would enhance early diagnosis of COPD. Tobacco smoking is the main recognized cause of COPD but the actions carried out by GPs to help a patient to give up smoking result still insufficient. The majority of GPs recognize spirometry as necessary to early COPD diagnosis, but the main obstacle pointed out to its wider use was the too long time for the spirometry to be performed. GPs’ main reason for prescribing a bronchodilator is dyspnea and bronchodilators preferably prescribed are LABA and LAMA. Control of patient’s adherence to therapy is mainly carried out by GPs checking the number of drugs annually prescribed or asking the patient during a control visit. Finally, about how many COPD patients GPs believe are in their group of assisted patients, a mean range of 25-40 patients was reported, that is consistently below the forecast based on epidemiological data and number of patients assisted by each GP.

**Conclusions:**

The results obtained with this project confirm the validity of this informal approach to professional education. Furthermore, this inquiry provided important insights about the general management of COPD and the process of integration between RS and GPs activities on this disease condition in the long run.

## Background

Chronic Obstructive Pulmonary Disease (COPD) is a respiratory pathological condition caused by a chronic abnormal response to noxious inhaled agents, mainly cigarette smoking, characterized by persistent and mostly irreversible airflow limitation determined by bronchial alterations (chronic bronchitis), small airways disease and parenchymal destruction (pulmonary emphysema), and associated with systemic complications and frequently occurring comorbidities (cardiovascular diseases, cancer, osteoporosis etc.) [1-3].

Noncommunicable diseases, among which COPD holds a prominent position, are responsible for more than 35 million deaths worldwide, a conspicuous rate that increased from 57% of total mortality in 1990 to 65% in 2010, and also sets a relevant burden in terms of disability-adjusted life-years (DALYs) [4-6]. Estimates of COPD prevalence in Europe are between 5 and 10% with a wide variation among different countries and in relation to the employed methodology, while the mortality rate is about 18 per 100,000 subjects per year [7].

Respiratory diseases are the third cause of death in Italy and COPD is responsible for about half of respiratory deaths when lung cancer is excluded [8]. On the basis of the National Institute of Statistics (ISTAT) data [1], about 5% of adult Italian males are allegedly affected by COPD and about 4% of women (i.e some 2,600,000 of Italian citizens). However Viegi et al. found that 9.9% of people between 25 and 45 years suffer from obstructive disorders while in the overall age-range of 25-73 years a percentage spanning from 11% to 40% of people are affected depending on which criterion is applied among European Respiratory Society (ERS), clinical criteria, or American Thoracic Society (ATS) ones [9]. This hypothesis is supported also by a study in the real world which demonstrated relevant underdiagnosis and undertreatment [10].

This respiratory disease represents also a heavy charge in terms of social and economic costs, especially when it has reached a more advanced severity with increased incidence of acute exacerbations and hospital admissions. At this stage, there is an unavoidable need for continuous use of bronchodilators, inhaled or systemic corticosteroids and antibiotics, long-term oxygen therapy, rehabilitative programs, and other treatments related to various comorbidities, mandatory to improve and maintain patient’s health status [11-13].

COPD presents with persistent cough, sputum production, dyspnea and decreased exercise tolerance, but these symptoms are often disregarded by patients, in the majority smokers, in less severe stages of the disease because considered as unavoidable consequence of smoking habits instead of evidence of a disease. In fact, several studies demonstrated that the patient may well experience symptoms but do not always report them to his General Practitioner (GP) [14,15]. Thus, in most cases the diagnosis of COPD is carried out when the disease has already reached a moderate-to-severe level both of airflow obstruction and symptoms [16-18].

Using questionnaires focused on smoking status and respiratory symptoms in primary care can favour the early detection of COPD [19] as well as the use of the cards of respiratory risk [20]. Both these methods can screen patients and address them to a spirometry in order to confirm the diagnosis of COPD [21]. Indeed, spirometry is performed in a low percentage of patients suspected to have COPD and the diagnosis is often based on clinical findings only [22]. Recent data from particularly committed GPs database show a percentage of COPD diagnosis backed by spirometries of 60% [23]. Office spirometry [24] has been proposed as a mean to increase the amount and the correctness of COPD diagnoses and it proven to be feasible by general practitioners (GPs). However many problems concerning time availability, competence to interpret the results, patient’s adherence to test, and other organizational factors have so far impaired a more widespread use of spirometry, that is an easy doing test, very important to qualify the diagnosis, and assess the grade of disease severity together with other clinical parameters like number of previous exacerbations, nutrition status, dyspnea score etc. [25]. Likely, a critical factor to extend the diffusion of spirometry in general practice would be to increase GPs believe of its utility for the diagnosis of COPD, as demonstrated in some studies [26].

Many international guidelines have been published in the last ten years [3]. Since 2011 there are Italian official guidelines from the Ministry of Health [1], which have been updated in 2013 by the three mayor respiratory scientific societies together with a GP scientific society [2]. All these documents give recommendations on how to improve the diagnosis and treatment of COPD patients and to control the costs of this disease. However, there are many proofs that guideline recommendations for management of COPD are not adequately followed in clinical practice both by GPs [27] and also by some respiratory specialists especially in relation to treatment [28,29]. In particular, the observed prevalence of COPD in records of GPs is notably lower than would be expected according to what found using health questionnaires and specific studies [30] and this means that a great part of patients are not diagnosed and consequently not treated. These missing diagnoses have been measured in Italy in situation applying the WHO-GARD strategy for diagnosis (and were shown to reach percentage as high as 70%) [31]. On the other hand, respiratory specialists mainly deal with severe or very severe COPD, often in hospital, having scarce possibility to manage less severely affected subjects who undergo the greater reduction of respiratory function and represent the more consistent part of COPD patients.

Such a situation clearly points out the need for a close cooperation between GPs and respiratory specialists to increase the knowledge of COPD risk factors, its clinical and functional peculiarities, especially in the initial stages, the differential characteristics from other respiratory obstructive diseases like asthma, and the correct management and follow-up. A better knowledge of the disease and a wider use of spirometry are critical to diagnose more patients still neglected, do it at an earlier stage and properly treat established COPD.

Moreover, once diagnosed, patients suffering from COPD in most cases (except, in some areas in Italy, people treated with long term oxygen therapy) do not experience no long term follow up of the disease and no offer of pulmonary rehabilitation [28] while they are poor compliant with the pharmacological treatment [32].

In the light of the above mentioned considerations, FISAR (the charity of reference of the Italian Interdisciplinary Association for Research in Lung Disease - AIMAR, advocating for respiratory health improvement) devised and realized a project, called *Pneumocafè* using an informal setting of meetings (i.e. non ECM events) between GPs and their respiratory specialists (RSs) of reference.

The aim of Pneumocafè project is to improve, through an innovative approach, the diagnosis and management of COPD at primary care level, increasing the awareness of issues pertaining to early diagnosis, adequate prevention and correct treatment of the disease.

## Methods

The project was based on a series of informal meetings to be held all over the Italian territory where a RS, after pointing out the main characteristics of COPD diagnosis and management, discussed in a colloquial manner with the attending GPs their current practice compared with what official guidelines suggest, analyzed the actual problems in diagnosing and managing COPD patients, and shared the possible solutions at the community level for a more correct approach to these issues.

In the space of 30-45 days 50 RS, selected as to cover all Italian territory, had to perform a mean of 5 meetings lasting about 2 hours, each including a group of 10 GPs working in their reference zone. Thus, at the end of the arranged period, each RS would have met about 50 GPs. Time and location of the meetings were scheduled in agreement with the GPs, generally in hospital clinic or in GP’s group surgery. In these meetings RSs, also starting from clinical cases reported by them or by attending GPs, faced many issues including patho-physiological mechanisms of bronchial obstruction, significance of clinical symptoms, patients’ phenotyping, and clinical approach to diagnosis and long-term treatment, and gave strength to messages concerning the importance of a timely diagnosis, proper long term treatment and the compliance to treatment.

RSs were recommended to present practical materials like representative spirometric graphs, questionnaires, and educational booklets, already sent them by the scientific board of the project. These tools represented the basis for a discussion with the GPs on site and then were given them to be delivered to patients in each surgery, so as to spread adequate information on a given territory.

A following phase of implementation of this educational process is planned, when GPs, made aware of the problems, will be able to screen suspect cases among their assisted people sending screened subjects to RSs to have the diagnosis confirmed.

At the end of each meeting GPs had to fill in a questionnaire arranged by the scientific board that included 18 multiple-choice questions concerning their approach to COPD management (Additional file 1).

RSs, after the cycle of meetings they held with GPs, were requested to fill in a quality questionnaire to express their comment on this new format, suitability of educational materials, level of GPs participation, possible interest to repeat similar meetings on the same or other topics of respiratory medicine.

The results of the analysis of these questionnaires are here presented; descriptive statistics is used to analyze the data, reported as percentage to the total, and as range of variability (min-max) when requested.

## Results

1, 964 questionnaires were returned from 49 RSs. 1,864 questionnaires out of those received (94.91% of the total) resulted properly compiled and form the object of the present analysis.

The 49 RSs, 37 males and 12 females, were distributed all over the Italian country and practiced their profession both in public and private hospitals and in territorial sanitary facilities.

**Figure 1:**
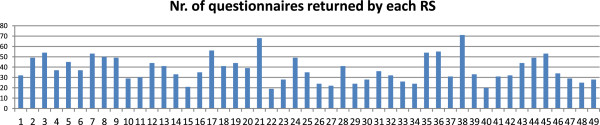
Number of questionnaires returned by each of 49 RS (horizontal axis: number of identification of RS; vertical axis: number of returned questionnaires).

**Figure 2:**
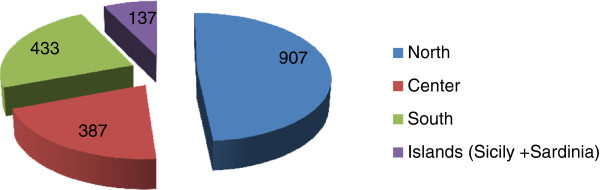
Geographic distribution of GPs and number of questionnaires received from each area.

GPs were 1,330 males (71.35%) and 534 females (28.64%), mean age 56,29 years (range 27-70 yrs). The provenience of participating RSs is reported in Additional file 2, while the number of questionnaires deriving from each center is reported in Additional file 3 and Figure 1.The geographical provenience of GPs along the Italian territory and the relative questionnaires are resumed in Figure 2. There is a marked prevalence of GPs practicing in north Italy (907 questionnaires), but also the other zone are well represented. Mean duration of general practice is 25.56 years (range: 0,5-40 yrs) with a mean of 1,302.43 patients assisted by each GP and 2,427,741 patients assisted in all.

To know which order of priority GPs give to pathological conditions that prevalently occur in their practice, and in particular to COPD, also to have an indirect measure of the prevalence of these disease, they were asked the question “From an epidemiological point of view which order of importance (1 = most important, 5 = least important) do you assign to the following diseases in the population of patients you assist?”. More than 60% of GPs answered that in their patients COPD has a mean-to-great prevalence (Table 1), preceded in terms of priority (score 1 + 2) only by diabetes and heart failure. Cancer and other chronic diseases seem less important according to their epidemiological burden.

**Table 1:** Epidemiological importance of COPD and other chronic diseases based on GPs clinical practice

Score*	**Heart failure**	**Diabetes**	**COPD**	**Cancer**	**Other chronic diseases**	**Total**
1	463	***840***	128	333	100	*1,864*
2	468	517	***583***	214	82	*1,864*
3	426	280	***763***	314	81	*1,864*
4	387	207	344	***771***	155	*1,864*
5	120	20	46	232	***1,446***	*1,864*
**Total**	*1,864*	*1,864*	*1,864*	*1,864*	*1,864*	

*1 = most important, 5 = least important.

In terms of impact COPD has on GPs practice it was scored as mean/high, similarly to heart failure, while the priority was again assigned to diabetes (Table 2).

**Table 2:** Answers of GPs to the question “How do you score these diseases in terms of impact on your practice?”

Score*	**Heart failure**	**Diabetes**	**COPD**	**Cancer**	**Other chronic diseases**	**Total**
1	458	***727***	159	421	99	*1,864*
2	498	509	***545***	249	63	*1,864*
3	414	379	***699***	284	88	*1,864*
4	385	224	388	***698***	169	*1,864*
5	109	25	73	212	***1,445***	*1,864*
**Total**	*1,864*	*1,864*	*1,864*	*1,864*	*1,864*	

*1 = most important, 5 = least important.

Subsequently, GPs were requested to indicate which COPD guideline they preferentially adopted in their clinical practice and the majority of them answered they were using GOLD guidelines (almost 62%), whereas almost a quarter of GPs affirmed they carried out their practice without using any guideline (Table 3).

**Table 3:** COPD guidelines GPs preferentially use in their clinical practice

**Type of Guideline**	**N**	**%**
GOLD guidelines	***1,150***	***61.70***
Guidelines of Italian Agency for Health Services (AGE.NA.S)	69	3.70
Guidelines of the Italian Respiratory and General Medicine Associations	230	12.34
No guidelines, following recommendations of pulmonary specialist	415	22.26
**Total**	*1,864*	*100*

**Figure 3:**
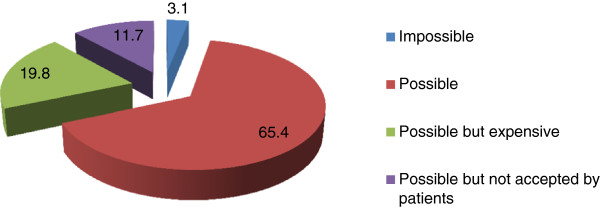
GPs’ opinion about an early COPD diagnosis among patients they assist.

Regarding early diagnosis of COPD and what in general should be done to improve it, the prevalent opinion of GPs (1,598 GPs, 85,73%) is that a screening on groups of patients at risk (active smokers or ex-smokers aged ≥ 40 years) might be a useful tool (Table 4).With reference to GPs’ individual practice, at the question whether an early diagnosis of COPD would be possible in the group of patients they assist, 65.4% answered positively even if some pointed to possible problems raised by costs or adherence by patients, whereas only 3.1% affirmed it was impossible (Figure 3).

**Table 4:** Proposals of GPs to reach early diagnosis of COPD

**Proposal**	**N**	**%**
Screening on general population over 40 years of age	266	14,27
Screening on groups of patients at risk among those they assist (ex- smokers or active smokers over 40 years of age)	***1,598***	***85,73***
**Total**	*1,864*	*100*

As to patient’s smoking habits, GPs should have updated records of their assisted, but only 52.90% of participating GPs declared to be able to always know how many smokers or ex-smokers are in their group of patients, whereas almost half of them were not informed on smoking habits of their assisted. GPs behave differently when dealing with a smoker and a personal care to help them to give up smoking is displayed only by about 30% of them (Table 5).

**Table 5:** Actions carried out by GPs to help a patient to give up smoking

**Actions**	**N**	**%**
I help him to give up smoking with a minimal advice (five “A”)	***606***	***32.51***
I address him to a specialized center for smoking cessation	479	25.70
I would address him to a specialized center for smoking cessation should there be any available	306	16.42
I only say him that he has to give up smoking	473	25.37
**Total**	*1,864*	*100*

GPs were also asked to express their opinion on what would be the cigarette amount exposing patients to the greatest risk for COPD and the majority (58.37%) answered 30 py (pack years = number of daily cigarette packs by number of smoked years), while 18.72% reported 20 py, 11.96% 10 py, and 10.95% affirmed they did not know.

With the aim of investigating the GPs knowledge about COPD exacerbations, another question was formulated regarding the GPs opinion about the statement that the majority of acute bronchitis episodes occurring in general practice are exacerbations of COPD, and this was agreed by 1,623 participants (87.07%).

Coming to the diagnosis of COPD, the majority of GPs recognize spirometry as necessary to diagnose the disease, while one third of them think it is necessary but not sufficient to reach the diagnosis and a very little percentage believe that COPD diagnosis must be based on clinical elements (Table 6).

**Table 6:** Answers of GPs to the question “How do you consider spirometry for the diagnosis of COPD?”

**Answer**	**N**	**%**
Necessary	***1,133***	***60.78***
Necessary but not sufficient	683	36.64
COPD diagnosis is only clinical	48	2.58
**Total**	*1,864*	*100*

In relation to spirometry availability to enhance the diagnosis of COPD in general practice, GPs mainly answered that having this facility in the health district of their reference would be the best solution, although a not negligible percentage of them affirmed the utility of a spirometer in their surgery (Table 7).

**Table 7:** GPs’ opinion about spirometer availability to favour the diagnosis of COPD

**Opinion**	**N**	**%**
Spirometer in own surgery	717	38.46
Spirometer in health district of reference	***825***	***44.26***
More spirometers in hospital	322	17.28
**Total**	*1,864*	*100*

Moreover, GPs have been asked what in their working place was the main obstacle to early COPD diagnosis and the main impediment pointed out in all districts of GPs provenience was the too long time to be waited for the spirometry to be performed (55.74%), followed by the cost patients have to sustain for this examination (17.97%) (Table 8).

**Table 8:** Main obstacles to early COPD diagnosis GPs encounter in their working places

**Obstacles**	**N**	**%**	**North**	**Center**	**South**	**Islands**
Spirometry availability too far	271	14.54	122	41	80	28
Cost of the examination	335	17.97	159	41	98	37
Too long time to be waited for spirometry	***1,039***	***55.74***	***523***	***247***	***208***	***61***
Lack of respiratory specialists in that district	219	11.75	103	58	47	11
**Total**	*1,864*	*100*				

As to the GPs ability to manage specific clinical cases, they were requested to choose among different possibilities inherent the diagnostic work-up of a patient 50 years old suspected of having COPD and presenting normal results of a simple (only forced expiratory volumes) spirometric examination. The majority of GPs (40.95%) revealed they would send such a patient to the respiratory specialist, while 28.75% would make patient to perform a global spirometry possibly also with a bronchodilation test (Table 9).

**Table 9:** What GPs would choose to do in a 50 yrs old patient suspected of COPD and presenting normal results with a simple spirometric test

**Choice**	**N**	**%**
Investigating an alternative diagnosis	175	9.40
Request a global spirometry	390	20,90
Request a global spirometry with bronchodilation test	536	28,75
Request consultation of a respiratory specialist	***763***	40,95
**Total**	*1,864*	*100*

In another clinical case, GPs were asked which is the first test they would perform in an old ex-smoker patient claiming of exertional dyspnea and the prevalent choice was a spirometric examination (sum of simple and global spirometry percentages), followed by ECG, cardiologist or pneumologist consultation (Table 10).

**Table 10:** First test GPs would perform in an old patient with exertional dyspnea

**Test**	**N**	**%**
ECG	374	20.06
Echocardiogram	115	6.17
Cardiologist consultation	408	21.90
Pneumologist consultation	391	20.97
Simple spirometry	348	18.67
Global spirometry	228	12.23
**Total**	*1,864*	*100*

**Figure 4:**
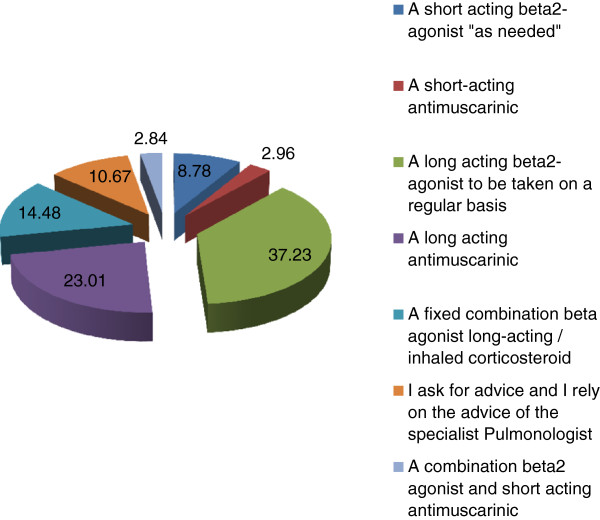
Bronchodilator drugs preferentially prescribed by GPs to a mild-to-moderate symptomatic COPD patient at first diagnosis.

**Figure 5:**
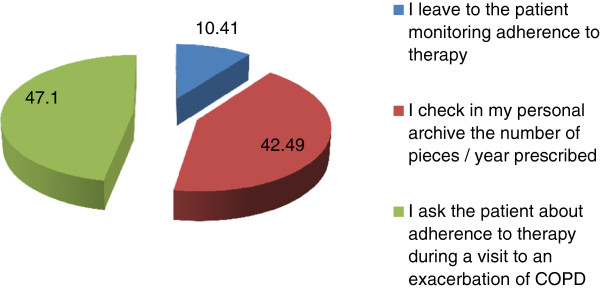
How GPs assess patient’s adherence to therapy.

Concerning the therapeutic approach to COPD, GPs’ main reason for prescribing a bronchodilator is dyspnea, followed by cough and sputum production, whereas the least importance is attributed to the patient’s capacity of performing daily activities (Table 11).Bronchodilator drug GPs preferentially prescribe to a symptomatic patient with mild-to-moderate COPD at first diagnosis is a long-acting beta-2 agonist taken on a regular basis, and the second preferred choice is a long-acting antimuscarinic drug (i.e LABA or LAMA are prescribed by more than 60% of GPs) (Figure 4).GPs were also asked whether and how they verify the adherence of their patients to the arranged therapeutic plan and the correct uptake of drugs. The majority of GPs (47.41%) answered they ask patients information about their adherence to therapy during a control visit, whereas another consistent percentage of GPs affirm they check in their records the number of drugs prescribed annually and only 10.41% of them let patients verify on their own the adherence to therapy (Figure 5).

**Table 11:** Reasons why GPs prescribe a bronchodilator drug

Score*	**Dyspnea**	**Level of bronchial obstruction at spirometry**	**Cough**	**Sputum production**	**Capacity of performing daily activities**	**Total**
1	**752**	713	54	20	325	*1,864*
2	**790**	509	176	117	272	*1,864*
3	168	317	***812***	178	389	*1,864*
4	34	214	641	***829***	146	*1,864*
5	120	111	181	720	***732***	*1,864*
**Total**	*1864*	*1864*	*1,864*	*1,864*	*1,864*	

*1 = most important, 5 = least important.

**Figure 6:**
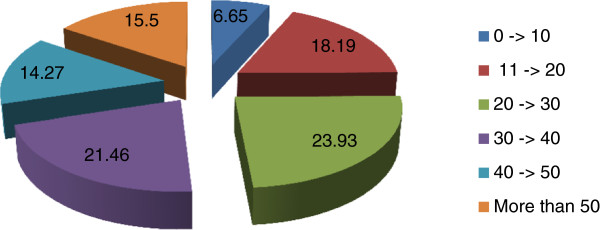
Number of COPD patients GPs estimate to be present in their group of assisted patients.

Finally, GPs were asked how many COPD patients they estimated to be in the whole group of patients they assisted (Table 12 and Figure 6), and a wide variability of prevalence was reported, the most frequent numbers in the whole group of GPs being 20 to 30 and 30 to 40 patients. However, in southern Italy there was a consistent number of GPs reporting they have more than 50 COPD patients. On the average the estimated number of COPD patients declared by GPs is consistently below the forecast in accordance with the epidemiological data and the number of patients assisted by each GP.

**Table 12:** Number of COPD patients GPs estimate to be present in their group of assisted patients

**N of patients**	**N of GPs**	**%**	**North**	**Center**	**South**	**Islands**
0 to 10	124	6,65	63	18	28	15
11 to 20	339	18,19	176	54	81	28
20 to 30	446	23,93	253	94	75	24
30 to 40	400	21,46	199	93	80	28
40 to 50	266	14,27	118	55	71	22
More than 50	289	15,50	98	73	98	20
**Total**	*1,831*	*100*				

The agreement of RSs towards such type of meetings has been recorded in a questionnaire, whose results are summarized in Tables 13 and 14.

**Table 13:** Results of the agreement questionnaire filled in by RSs (results are expressed as percentage on the whole group of RSs)

**Issue**	**Excellent**	**Good**	**Sufficient**	**Insufficient**
Relevance of treated topics for educational purposes	66.0	34.0		
Level of participation/interest of GPs	41.5	55.0	3.5	
Quality of educational material	83.0	14.0	3.0	
Impact of the event on RS’s daily practice	34.5	55.0	10.5	
Evaluation of this novel format	48.0	38.0	10.5	3.5

**Table 14:** Opinions of RSs about repeatability of events with this format (results are expressed as percentage on the whole group of RSs)

**Question**	**Yes**	**No**	**Don’t know**
Do you think GPs are interested in participating in other similar events on different respiratory topics?	93.0		7.0
Are you interested in participating in another educational event like *Pneumocafè*?	93.0		7.0

## Discussion

COPD is a frequently encountered chronic disease condition in primary care, however the official prevalence data underestimate the real burden of the disease that often is recognized only when has already reached an advanced stage [14,16], such as in a not negligible number of patients in the community the disease is not diagnosed and effective treatment not delivered or delayed.

Besides unrecognizing symptoms suggestive for COPD, spirometry is underutilized in general practice [33,34], and in Italy at least 30% GPs do not use spirometry to diagnose COPD because they consider this examination not necessary or claim of logistical limitations [35].

Therefore a better approach to COPD diagnosis and management in general practice appears mandatory to decrease the rate of underdiagnosis and undertreatment, and in this context is crucial the cooperation between RSs and GPs.

The unusual format of professional education devised in Pneumocafè Project, as results from the RSs’ answers to quality questionnaire, has been agreed by the wide majority of them, who believe this event is also an opportunity to know other GPs or reinforce the relationships with those already known, also receiving from them new patients to study because suspected of having respiratory diseases. Furthermore, this colloquial format without slides has been recognized by RSs as an efficacious tool to transfer scientific information to GPs, who endorse it much more than in a classic congress. The quality of educational material has been mostly believed as suitable for the purposes of the event, however some RSs would also have multimedial materials to reach a greater interactivity with the participants, while other RSs suggest a preliminary collection of GP’s needs (topics, case reports and other) such as to set the meeting on a higher level of interactivity.

One of the most distinctive and valuable characteristic of the present inquiry is represented by the high number of GPs (assisting more than 2 millions of patients in all) attending the meetings, whose wide distribution across the Italian territory gives particular truthfulness and reliability to the results. In fact, although this project was not designed to assess the epidemiological prevalence of COPD using field interviews or other instruments, it nevertheless allows to draw a comprehensive view of what occurs in real life relatively to COPD diagnosis and treatment, valuable to plan future initiatives aimed at improving the present situation. The greater number of GPs from Northern Italy in this study is accounted for by the larger population and consequently the greater presence of GPs and RSs in this area of the Country.

According to the belief of GPs participating this investigation, the epidemiological burden attributable to COPD in their experience is not negligible, lower than diabetes but equal to chronic heart disease. In this regard, several studies demonstrated that COPD patients are at increased risk of cardiovascular morbidity and mortality [36,37] and the cardiovascular comorbidity can often represent a priority in the management of the patient. On the other hand, among patients admitted to hospital for chronic heart failure the presence of COPD has been found in about 35%, but only 43% of them reported to have COPD, whose one-third had the diagnosis of COPD confirmed by spirometry [38,39]. As to concerns the impact on their practice that GPs attribute to diabetes, the prevalence of this disease in Italy has recently estimated to be 4.9% [40], apparently not different from that of COPD. However, the diagnostic approach to diabetes is easier and based on simple parameters like blood glycemia or glicated haemoglobin, thus the estimated prevalence of this disease likely approximates reality more than that of COPD. In addition, specialized clinics for diabetes supporting general practice are much more numerous and more organized than respiratory services in Italy.

In the present inquiry GOLD guidelines [3] are reported to be referred at by over 60% of GPs. GOLD has been formed in 1998: since then various updated editions of this initiative followed and also Italian translations have been published in recent years. National institutional documents [1,2] drawn to adequate and uniform the approach to COPD by Italian physicians have been published more recently, especially the last one [2]. Due to recent diffusion of these documents, it is probable that GPs are not completely aware of this availability yet. Besides, if one GP already got used consulting a given guideline, moreover the only available to him for a long time and with which he gained some familiarity, it is possible that he can have some difficulties to change his reference. Use of guidelines to increase COPD diagnoses is still widely discussed, even if recent investigations report that the more updated guidelines can identify greater proportions of patients in the mildest and most severe categories, however without avoiding discordances between airflow limitation severity and exacerbation risk [41]. Instead, it seems difficult to find the reasons why over 20% of GPs do not consult any guideline but only refer to RS. This could be they do not know the guidelines, or better they have good and easy relationships with the reference RS. Alternatively, they might be not confident at all in guidelines, or do not want to engage themselves in a consultation that is time-consuming and does not always help to work out specific problems but only give general recommendations to be applied in each clinical context. However, it is a general observation that, in spite of progressive improvement of evidence-based guidelines production and dissemination, their awareness is not sufficient and their use for day-to-day care is still scarce [42], also because guidelines are generally developed by experts who pay more attention to methodological rules and quality grades than to the objective of integrating scientific evidences into practice [43]. The inclusion of GPs in drawing up guidelines is important to achieve sufficient suitability for application in “real-life” and this is the reason why the recent national document on COPD diagnosis and management [2] has provided the participation of a GP’s scientific society under the control of Italian Ministry of Health. In addition, it is not sufficient to disseminate the guidelines on a national basis to reach an acceptable level of application, but they should be also implemented at regional and local level. In this respect, some projects have been devised to implement the knowledge of guidelines among GPs, based on multifaced knowledge translation interventions consisting of interactive education, quality circles and practice-based tools in primary care [44]. Some projects consider useful to increase dissemination of guidelines, like the “Knowledge to Action” (KA) [45] that recommends using tailored and comprehensive approaches at different level (doctor, team practice, hospital etc.) after assessing local barriers, available resources and other practical aspects, and in some countries institutions adopting specific KA processes have been charged with the implementation of respiratory guidelines [46]. However, a critical review of the results of theories so far adopted in many studies for this purpose points out they are not satisfying and the approach needs further improvement [47,48]. In our country this task is mainly carried out by respiratory scientific associations and Pneumocafè project was just designed to comply with this need.

Early detection of COPD, so as of other respiratory diseases like lung cancer, is a significant issue [49] as it would increase finding patients affected by the disease and also favour the possibility of undertaking efficacious preventive and therapeutical measures, thus improving the long-term prognosis of these patients [15,50]. However, this represent a worldwide problem [51], since at the beginning of the present century 10 million physician-diagnosed COPD diagnoses have been reported in U.S.A., that is much less than what expected based on the 24 million adults with impaired lung function according to NANHES III survey [52]. Similarly, in a Dutch Survey [53] the prevalence of self-reported COPD was almost twice as high as the prevalence based on GP registration. This has also heavy economic implications because the cost of advanced and severe forms of COPD is as much as seven times higher than that of mild cases [54,55].

Obviously, COPD underdiagnosis and undertreatment mainly relate to the mild stages of the disease and the causes may be various. Some authors [14] recognize as a crucial factor the need for more evidence of beneficial effects of interventions on patients with mild symptoms, because if this is true the approach can become cost-effective. More likely, the reasons for underdiagnosis should be attributed both to the patients and to the physicians, because 33% of patients at high risk of COPD and presenting symptoms never consulted their physician. On the other hand, among COPD patients who consulted the physician only 60% performed spirometry [55]. Thus, a better knowledge of COPD symptoms must be enhanced not only among GPs, but also in general population, as well as a more widespread use of spirometry as a tool to functionally qualify clinical symptoms and/or to reveal possible functional alterations in subjects not presenting symptoms [56,57]. A recent retrospective analysis [58] of a wide cohort in UK revealed many neglected opportunities in routine care to make an early diagnosis of COPD in patients with lower respiratory tract symptoms and several long-term comorbidities.

In this inquiry the great majority of participating GPs confirmed the importance of early COPD diagnosis and they think the best method to accomplish this purpose would be to perform a screening among their assisted patients who are at risk for this disease, namely smokers or ex smokers aged 40 years or more. The majority of GPs think that this task is feasible in their clinical practice, although at least 30% of them raise some difficulties relative to the costs and the patient’s acceptance of such screening.

As for where the spirometry could be delivered, the majority of GPs point to the health district of reference while a percentage as high as 38% think own surgery as the best setting.

However, a previous Italian study on symptomatic patients only showed that spirometry, while certainly feasible in the general medicine setting, encounters poor enthusiasm in GPs (mainly due to lack of time) who reported also non-negligible problems regarding the maintenance of adequate standards of performance and interpretation [24].

A valid support to improve early diagnosis of COPD might be the use of questionnaires especially in practice-managed conditions [19,21], while other experiences [59,60] point out the validity of making an early diagnosis of COPD through smoking cessation programs.

The common remark that spirometry is performed much less frequently than what would be necessary [22,35,51,55] could be related to the fact that GPs do not believe necessary this examination and base their diagnosis only on clinical issues. Indeed, this does not seem the true or the main reason because in the present survey it emerged that almost two thirds of GPs believe spirometry necessary to diagnose COPD and only less than 3% think COPD diagnosis is only clinical. Thus, it is conceivable that spirometry is not sufficiently available in general practice at present and should be further provided both in terms of number of facilities that can perform it with an easy access, and in terms of greater familiarity with this examination, ability to interpret the results, and availability to perform it in one’s own office. In fact, in this inquiry GPs expressed with the same percentage the wish to have more spirometers in the health district of their reference or a spirometer in their surgery. The main reason GPs point out for the lack of spirometric examinations is the too long time awaited for the spirometry to be performed. However, there is still a general belief in general practice that implementation of spirometry is complicated [35,56] and that training of GPs for interpreting spirometry is only a part of the solution [57], so as the better intervention would be to increase spirometry services [34].

All these considerations urge to better arrange the respiratory health framework all along the Italian territory, by incentivizing the possibility of performing spirometry and the relationships between GP and RS for a continuity of care to patients affected with COPD. This emerged also from the behaviour of general practitioners about the clinical cases they have been asked to manage in this inquiry, where the consultation of a respiratory specialist has been the measure mostly requested.

The tobacco epidemic has now become a global phenomenon, and it represents a problem also in our country, where smoking has a prevalence of 26.2% among adults (30.0% in males and 22.2% in females) [61] and smoking related diseases are responsible for 10% of deaths of adult population. Active smoking undeniably is the main avoidable cause of morbidity and mortality, and the main risk factor for COPD, lung cancer and cardiovascular diseases. Thus, it is very important to realize that effective interventions to help smokers cease their habitude and to convince non-smokers not to start smoking. Furthermore, tobacco smoking is not only the main cause of many respiratory disease conditions, but also a cause of worsening of an established respiratory disease. On the contrary, quitting smoking slows down the progression of COPD towards more severe levels of disease and invalidity. For these reasons chest physicians have been invited to consider smoking cessation as an essential therapeutic measure, the first provision to give the active smoker at the very moment of the first diagnosis of COPD [1,62]. A person whom a smoking related disease has been diagnosed and who still is an active smoker should be considered a “hard-core smoker” [63]. These kind of smokers should be treated with an “intensive treatment”, far more intensive than the so-called “minimal advice” (or 5As method) [64], which can be delivered only by specifically trained staff in a specialized setting. In our survey, only one out of four GPs refers such patients to the proper treatment (a specialized centre for smoking cessation); the remainders (i.e. the majority) are divided into: one other quarter which does almost nothing (only “recommending” to quit), one third which delivers a minimal advice and another 16% which would refer the patient to a smoking cessation clinic, were this latter available. It then comes that only a minority of COPD smokers can actually receive the proper treatment they deserve.

COPD is characterized by persistent airflow limitation and lung hyperinflation both in static and dynamic conditions, and the main symptom patients claim about is exertional dyspnea, that in the most severe cases can also make impossible the normal daily activities. Therefore, bronchodilators, especially the long-acting ones, both beta-adrenergic and antimuscarinic [65-68], are the cornerstone of the long-term treatment of this disease, because they decrease the airways resistances and lung hyperinflation and improve symptoms, exercise tolerance and reduce the rate of acute exacerbations also improving patient’s daily life and slowing up the disease progress. It has been demonstrated [69,70] that early morning is the worse period of the day for COPD patients, thus an inhaled bronchodilator capable of inducing a rapid bronchodilation and maintaining it for 24 hours, is a real advantage for these patients and increases their adherence to therapy. A significant correlation has been demonstrated after bronchodilator use between the decrease in inspiratory capacity and the reduction of dyspnea level [71]. Moreover, it is important that the inhaler be user-friendly and characterized by a low internal resistance so as to be effectively used also with low inspiratory flows. In conformity with these pathophysiological and clinical issues, in this inquiry the leading reason for GPs prescription of bronchodilators in their COPD patients is just because they complain about dyspnea and present a marked grade of airflow limitation at spirometry, and the long-acting bronchodilators are mainly prescribed (more than 60% of GPs).This is in accordance with the evidence-based guidelines that recommend the use either of a LABA or of a LAMA as first choice for the long-term treatment of COPD, while the two bronchodilators can be used together if necessary. In fact, two bronchodilators with different modality of action on bronchial tone may be in some patients more suitable than adding corticosteroids to decrease the lung hyperinflation and increase exercise tolerance, and in this context various combination of different bronchodilators are now ready to be introduced in clinical practice [72].

A non negligible percentage of GPs (about 12%) prescribe only short-acting bronchodilators (SABA) as needed to COPD patients. This can be interpreted that the majority of their patients have a mild disease, although a beneficial effect has been demonstrated in COPD with a long-term treatment beginning in the first phases of the disease, where therapy can prevent the functional decline, much more marked at this stage, and significantly modify the outcome [73,74]. The GPs who prescribe only SABA in COPD seem to have insufficient knowledge of pathophysiological aspects of the disease or alternatively mainly look at the costs of therapy that are lower with SABA than with Long-acting bronchodilators, although SABA should be used only during acute exacerbations as rescue therapy, as extensively recommended in COPD guidelines [1-3]. More than 14% of GPs affirm to prescribe a fixed combination of LABA and ICS (inhaled corticosteroids). Such an approach does not seem to comply completely with the recommendations of guidelines (GOLD included) according to which this choice should be kept for most severe patients showing a high annual incidence of exacerbations or for COPD patients with an asthmatic comorbidity. We have no data on how many Italian COPD patients could be included in these two sub-populations, and the type and severity of COPD patients have been demonstrated to vary in different clinical series, even if an exacerbator phenotype has been identified among COPD patients [75]. Therefore, we can draw no conclusion about the appropriateness of this prescription, and generally speaking a certain over prescription of LABA/ICS combination can be supposed.

It is axiomatic that a certain therapy results beneficial to patients provided that they take the drugs and do it correctly, that is they are adherent to treatment, and the practice of complying with a medication regimen must be considered as important as the effect yielded by the medication [76]. In fact, a lower adherence to treatment can cause marked worsening of health status [77], whereas the adherence to inhalation therapy in COPD is associated with a lower risk of death and hospitalization for COPD exacerbations [78]. The adherence to treatment in COPD takes advantage of a simplification of therapeutical regimen by reducing the number of doses, and of the prescription of user-friendly inhalers and rapidly acting drugs whose effect can be perceived by the patient [79,80].

Adherence to therapy, however, cannot be assessed only at the beginning of treatment, but must be checked periodically to have the certainty that the patient takes the drugs and does it correctly, but the ways to do this are not codified and often let to the individual initiatives. In this respect, the majority of GPs in the present inquiry affirm they ask patients information about their adherence to therapy during a control visit, whereas a little lower percentage of GPs declare to check in their records the number of drugs they prescribed to the patient during the year. These control initiatives does not assure that patients really took the drugs in a given period of time, even if the clinical course of the disease in the same period can be an indirect indicator of whether the drug has been taken or not. In a survey on more than 25 thousands COPD patients, those who were taking combination LABA/ICS (inhaled corticosteroids) had better adherence than those taking jointly the single drugs and adherence was better for a once daily (OD) agent than a multiple-dose-per-day agent while it was less associated with patient’s level or comorbidities than with drug delivery factors [81].

The last question GPs were asked to answer in this inquiry was inherent to the number of COPD patients they estimated to be among the own group of assisted patients. Due to a great dispersion of estimates, it has not been possible to establish with certainty a mean number of COPD patients, however the ranges between 20 to 30 and 30 to 40 patients have been chosen by the higher number of GPs without clear differences according to geographic residence. Thus it seems likely that 30 is more or less the number that the majority of GPs believe representing the COPD amount among their group of assisted patients. These data are a confirmation of COPD underdiagnosis, because according to a mean number of 1,300 assisted patients per GP and a 5% prevalence of COPD in adult population, each GP should count on the average 50-75 COPD patients among their assisted.

## Conclusions

This Pneumocafe’ project was aimed at providing data about COPD management in a general practice as well as at testing a new and more informal approach to professional education and our results, in our opinion, confirm the validity of this approach. This non academic opportunity seems very suitable to understand and solve some doubts and possibly to remove obstacles not allowing a correct management. Besides, the present study provided important insights about the general management of COPD as a chronic disease condition and about the process of integration between RS and GPs activities on this condition in the long run.

A general conviction has emerged that the awareness about COPD, especially when it is at an early stage, should be increased, not only among GPs but also in general population. For this purpose the active participation of GPs in these meetings can warrant a wider spread of COPD knowledge among the general population which should pay more attention to the often neglected symptoms of the disease, the damages it can determine if not treated and the ways to avoid them.

Finally, an improvement of knowledge of the characteristics and activities of the management of COPD appears to be necessary after our results.

Even if spirometry is recognized to have a fundamental role for getting an early diagnosis of COPD (consequently allowing interventions aimed at preventing its worsening) our results showed that this examination is not sufficiently available or carried out. Most GPs recognize that it should be diffused to a greater extent and this requirement appears as a compelling commitment not only for the physicians themselves but also for the institutional health officers, responsible for a more efficacious organization of this field.

Also elements necessary for the management of the disease, like smoking cessation and pharmacologic treatment after diagnosis seem to need to be refreshed, possibly through a dissemination of Italian Guidelines.

This survey will be repeated in a couple of years after interventions to measure the change.

## Competing interests

The authors declare that they have no competing interests.

## Supplementary Material

Additional file 1 The GP’s Questionnaire. Click here for file

Additional file 2 Provenience of Respiratory Specialists (RS). Click here for file

Additional file 3 Number of Questionnaires returned by each RS. Click here for file
