# Supplementary material for: Pneumocafé project: an inquiry on current COPD diagnosis and management among General Practitioners in Italy through a novel tool for professional education
Source: Multidiscip Respir Med. 2014 Jun 12;9(1):35. doi: 10.1186/2049-6958-9-35 (PMC4061438; doi:10.1186/2049-6958-9-35)
Supplement: Additional file 1 — The GP’s Questionnaire. [file 2049-6958-9-35-S1.doc]

**Appendix 1: The GP’s Questionnaire**

General Informations

GP Age: __________________ Sex: M □ F □

Years of GP: ______________________

Patients N.: ______________________

**Questionnaire**

1. **From an epidemiological point of view, I assign this priority rank to the following diseases in the population of patients I assist** (1= most important ; 5= least important) :

- Heart failure
- Diabetes
- COPD
- Tumors
- Other chronic diseases

1. **Concerning the impact on my clinical practice, I assign this priority rank to the following diseases in the population of patients I assist** (1= most important; 5= least important) :

- Heart failure
- Diabetes
- COPD
- Tumors
- Other chronic diseases

1. **The COPD guidelines whose I refer to in my clinical practice preferably are** *(only one answer)):*

- GOLD guidelines
- AGENAS guidelines
- Guideline published by respiratory scientific societies in cooperation with a GPs’ scientific society
- I do not refer to any guideline and follow specialist’s advice

1. **For early COPD diagnosis I would recommend** *(only one answer):*

- Screening of general population aged more than 40 years
- Screening of selected populations of assisted patients (ex-smokers or active smokers aged more than 40 years)

1. **I believe that early COPD diagnosis in my assisted patients at risk would be in my setting** *(only one answer):*

- Impossible
- Possible
- Possible but expensive
- Possible but not agreed by patients

1. **May I always retrieve from my records how many smokers or ex-smokers are in my assisted population ?** *( only one answer ):*

- Yes
- No

1. **If one of my assisted patients affected with COPD is an active smoker** *(only one answer)*:

- I help him to give up smoking with a minimal advice (five “A”)
- I address him to a specialized centre for smoking cessation
- I would address him to a specialized center if they would be available
- I only tell him to give up smoking

1. **Are at greater risk of COPD subjects smoking** *(only one answer):*

- 10 pack/years
- 20 pack/years
- 30 pack/years
- Don’t know

1. **Many acute bronchitic episodes treated ( usually with antibiotics) in general practice really are symptoms of COPD** (*only one answer):*

- True
- False

1. **For COPD diagnosis spirometry is** (*only one answer) :*

- Necessary
- Necessary but not sufficient
- COPD diagnosis is clinical only

1. **To favour COPD diagnosis I think would be more useful** *(only one answer):*

- A spirometer in my office
- A spirometer in my reference district
- A spirometer more in hospital

1. **In my setting, the main obstacle to early COPD diagnosis is** (*only one answer) :*

- Spirometry too far
- Ticket cost
- Too long time to wait for spirometry
- Lack of specialists within reach

**An adult 50-years-old subject , who I suspect to be affected with COPD, presents normal results at simple spirometry** (*only one answer) :*

- I investigate an alternative diagnosis
- I ask for a global spirometric examination
- I ask for a global spirometric examination with a pharmacodynamic reversibility test
- I ask for a specialistic respiratory consultation

1. **An old , ex-smoker, patient of mines complains of exertional dyspnea . As first investigation I arrange** *(only one answer):*

- Electrocardiogram
- Echocardiogram
- Cardiologic specialistic consultation
- Respiratory specialistic consultation
- Simple spirometry
- Global spirometry

1. **My prescription of bronchodilator drugs is based on** (1= most important ; 5= least important):

- Dyspnea
- Degree of bronchoial obstruction at spirometry
- Cough
- Spurum production
- Ability to perform daily activities

1. **The bronchodilator drug I firstly prescribe to a symptomatic patient recently diagnosed affected with mild-to-moderate COPD is** *(only one answer)*:

- Short-acting beta-2 agonist (SABA) as needed
- Short-acting antimuscarinic (SAMA) drug
- Long-acting beta-2 agonist (LABA) to be taken regularly
- Long-acting antimuscarinic (LAMA)
- Fixed combination LAMA/ICS (inhaled corticosteroid)
- Combination of SABA and SAMA
- I ask for a respiratory specialist’s consultation and follow his advice

1. **When bronchodilator drug has been prescribed chronically**
   *(only one answer)*:

- I let patient to control treatment adherence
- I check in my records the number of drugs annually prescribed
- I ask patient informations about adherence to therapy during a visit for COPD exacerbation

1. How many COPD patients are in your assisted population ? *(only one answer)*:

- From 0 to 10
- From 11 to 20
- From 20 to 30
- From 30 to 40
- From 40 to 50
- More than 50
