# Supplementary material for: Pneumocafé project: an inquiry on current COPD diagnosis and management among General Practitioners in Italy through a novel tool for professional education
Source: Multidiscip Respir Med. 2014 Jun 12;9(1):35. doi: 10.1186/2049-6958-9-35 (PMC4061438; doi:10.1186/2049-6958-9-35)
Supplement: Additional file 2 — Provenience of Respiratory Specialists (RS). [file 2049-6958-9-35-S2.doc]

**Appendix 2: Provenience of Respiratory Specialists (RS)**

| **RS Id*** | **City** | **Area** |
| --- | --- | --- |
| 1 | Brescia | North Italy |
| 2 | Salerno | South Italy |
| 3 | Cuneo | North Italy |
| 4 | Imperia | North Italy |
| 5 | Chivasso (TO) | North Italy |
| 6 | Catania | Sicily + Sardinia |
| 7 | Cesena | North Italy |
| 8 | Eboli (SA) | South Italy |
| 9 | Battipaglia (A) | South Italy |
| 10 | Passirana di Rho (MI) | North Italy |
| 11 | Vittorio Veneto (TV) | North Italy |
| 12 | Pozzuoli (SA) | South Italy |
| 13 | Frosinone | Middle Italy |
| 14 | Roma | Middle Italy |
| 15 | Cosenza | South Italy |
| 16 | Pisa | Middle Italy |
| 17 | Pescara | Middle Italy |
| 18 | Siracusa | Sicily + Sardinia |
| 19 | Caserta | South Italy |
| 20 | Piacenza | North Italy |
| 21 | Orbassano (TO) | North Italy |
| 22 | Bergamo | North Italy |
| 23 | Avellino | South Italy |
| 24 | Fano (PU) | Middle Italy |
| 25 | Messina | Sicily + Sardinia |
| 26 | Cagliari | Sicily + Sardinia |
| 27 | Tradate (VA) | North Italy |
| 28 | San Severo (FG) | South Italy |
| 29 | Ferrara | North Italy |
| 30 | Sesto San Giovanni (MI) | North Italy |
| 31 | Prato | Middle Italy |
| 32 | Bergamo | North Italy |
| 33 | Grosseto | Middle Italy |
| 34 | Vicenza | North Italy |
| 35 | Frattamaggiore (NA) | South Italy |
| 36 | Padova | North Italy |
| 37 | Roma | Middle Italy |
| 38 | Bologna | North Italy |
| 39 | Lodi (LO) | North Italy |
| 40 | Civita Castellana (VT) | Middle Italy |
| 41 | Roma | Middle Italy |
| 42 | Rozzano (MI) | North Italy |
| 43 | Verona | North Italy |
| 44 | Legnago (VR) | North Italy |
| 45 | Napoli | South Italy |
| 46 | Genova | North Italy |
| 47 | Terni | Middle Italy |
| 48 | Gorizia | North Italy |
| 49 | Novara | North Italy |

*RS Id = Respiratory Specialist identification number
