# Supplementary material for: Pneumocafé project: an inquiry on current COPD diagnosis and management among General Practitioners in Italy through a novel tool for professional education
Source: Multidiscip Respir Med. 2014 Jun 12;9(1):35. doi: 10.1186/2049-6958-9-35 (PMC4061438; doi:10.1186/2049-6958-9-35)
Supplement: Additional file 3 — Number of Questionnaires returned by each RS. [file 2049-6958-9-35-S3.doc]

**Appendix 3: Number of Questionnaries returned by each RS**

| **RS Id*** | **N. Questionnaries** |
| --- | --- |
| 1 | 32 |
| 2 | 49 |
| 3 | 54 |
| 4 | 37 |
| 5 | 45 |
| 6 | 37 |
| 7 | 53 |
| 8 | 50 |
| 9 | 49 |
| 10 | 29 |
| 11 | 30 |
| 12 | 44 |
| 13 | 41 |
| 14 | 33 |
| 15 | 21 |
| 16 | 35 |
| 17 | 56 |
| 18 | 41 |
| 19 | 44 |
| 20 | 39 |
| 21 | 68 |
| 22 | 19 |
| 23 | 28 |
| 24 | 49 |
| 25 | 35 |
| 26 | 24 |
| 27 | 22 |
| 28 | 41 |
| 29 | 24 |
| 30 | 28 |
| 31 | 36 |
| 32 | 32 |
| 33 | 26 |
| 34 | 24 |
| 35 | 54 |
| 36 | 55 |
| 37 | 31 |
| 38 | 71 |
| 39 | 33 |
| 40 | 20 |
| 41 | 31 |
| 42 | 32 |
| 43 | 44 |
| 44 | 49 |
| 45 | 53 |
| 46 | 34 |
| 47 | 29 |
| 48 | 25 |
| 49 | 28 |
| ***Total*** | ***1864*** |

*RS Id = Respiratory Specialist identification number
